# Displacement of Spleen

**Published:** 1875-01

**Authors:** 


DISPLACEMENT OF SPLEEN.—* * * A lady, thirty-five years old, who had always been healthy, was injured by a fall from a wagon, which produced great pain in the left hypochondriac region, vomiting, and inability to move. The attending physician found a tumor at the site of pain, to which he applied leeches and friction, with mercurial ointment, but the pain and vomiting continued.

The writer was called in to see her about six weeks after the accident occurred. On examination he found a somewhat movable, circumscribed tumor, in the left iliac region, six inches in length and three and a half inches in breadth. Pulse 80; temperature normal. He diagnosed the tumor to be the spleen, and returned it to the left hypochondriac region, retaining it there by a compress and a firm binder.

After the reduction the pain and vomiting subsided. A few days later the patient was able to retain food and to leave her bed, to which she had been confined ever since the accident.—*Medical Examiner.*

